# NucleoFind: A Deep-Learning Network for Interpreting Nucleic Acid Electron Density

**DOI:** 10.1063/4.0000831

**Published:** 2025-10-27

**Authors:** Jordan S Dialpuri, Jon Agirre, Kathryn D Cowtan, Paul D Bond

**Affiliations:** University of York, York, North Yorkshire, United Kingdom

## Abstract

Nucleic acid electron density interpretation after molecular replacement remains a difficult problem for computer programs to deal with. Programs tend to rely on time-consuming and computationally exhaustive searches to recognise characteristic features. We present NucleoFind, a deep- learning-based approach to interpreting and segmenting electron density. Using an electron density map from X-ray crystallography, the positions of the phosphate group, sugar ring and nitrogenous base group can be predicted with high accuracy. On average, 86 % of phosphate atoms, 90 % of sugar atoms and 90 % of base atoms are located by the deep learning model in maps produced following protein molecular replacement. The wealth of context these predicted maps provide can then be used to automatically build more accurate and complete nucleic acid molecular models in a short amount of time.
